# Hearing and Seeing Nerve/Tendon Snapping: A Systematic Review on Dynamic Ultrasound Examination

**DOI:** 10.3390/s23156732

**Published:** 2023-07-27

**Authors:** Carmelo Pirri, Nina Pirri, Carla Stecco, Veronica Macchi, Andrea Porzionato, Raffaele De Caro, Levent Özçakar

**Affiliations:** 1 Department of Neurosciences, Institute of Human Anatomy, University of Padova, 35121 Padova, Italy; carla.stecco@unipd.it (C.S.); veronica.macchi@unipd.it (V.M.); andrea.porzionato@unipd.it (A.P.); rdecaro@unipd.it (R.D.C.); 2 Department of Medicine—DIMED, School of Radiology, Radiology Institute, University of Padua, 35122 Padova, Italy; nina_92_@hotmail.it; 3 Department of Physical and Rehabilitation Medicine, Hacettepe University Medical School, 06100 Ankara, Turkey; lozcakar@yahoo.com

**Keywords:** soft tissue, popping, subluxation, dislocation, ultrasonography, anatomy

## Abstract

Nerve/tendon snapping can occur due to their sudden displacement during the movement of an adjacent joint, and the clinical condition can really be painful. It can actually be challenging to determine the specific anatomic structure causing the snapping in various body regions. In this sense, ultrasound examination, with all its advantages (especially providing dynamic imaging), appears to be quite promising. To date, there are no comprehensive reviews reporting on the use of dynamic ultrasound examination in the diagnosis of nerve/tendon snapping. Accordingly, this article aims to provide a substantial discussion as to how US examination would contribute to ‘seeing’ and ‘hearing’ these pathologies’ different maneuvers/movements.

## 1. Introduction

Snapping commonly occurs as a result of the sudden displacement of an anatomical or pathological structure during the movement of an adjacent joint [1]. Apart from causing curiosity, the clinical scenario can often be accompanied by discomfort or pain, limiting daily professional/sporting activities [1]. Snapping is usually audible and palpable, but rarely visible; therefore, imaging and detecting the actual/responsible structure is crucial, but difficult as well [2,3,4]. Although radiographs, computed tomography and magnetic resonance imaging (MRI) are used for assessing several anatomic structures in this aspect, ultrasound (US) examination appears to be superior and able to contribute more [3,4]. Apart from its high resolution as regards nerve/tendon imaging, the dynamic evaluation of the structures in a patient- and physician-friendly approach is paramount for better understanding ‘snapping’ [2,3,4]. US examination provides a precise (real-time) correlation between the symptoms and the movement of the suspected structure [1,5,6]. Depending on the suspected nerve/tendon, snapping can be triggered/tested during any position, also conveniently reassuring the patient [5]. Prompt dynamic US examination requires excellent knowledge on US physics, and consequently regarding various artifacts and interactions with different tissues, for better interpretation of the US images/videos [2,3,4].

Although the use of US examination for snapping is well-known, there is no comprehensive review present in the pertinent literature describing how sonographic ‘seeing’ or ‘hearing’ can be performed. As such, the purpose of this article was to report the significance/utility of US examination in the diagnosis of snapping tendons and nerves.

## 2. Materials and Methods

**Figure 1:**
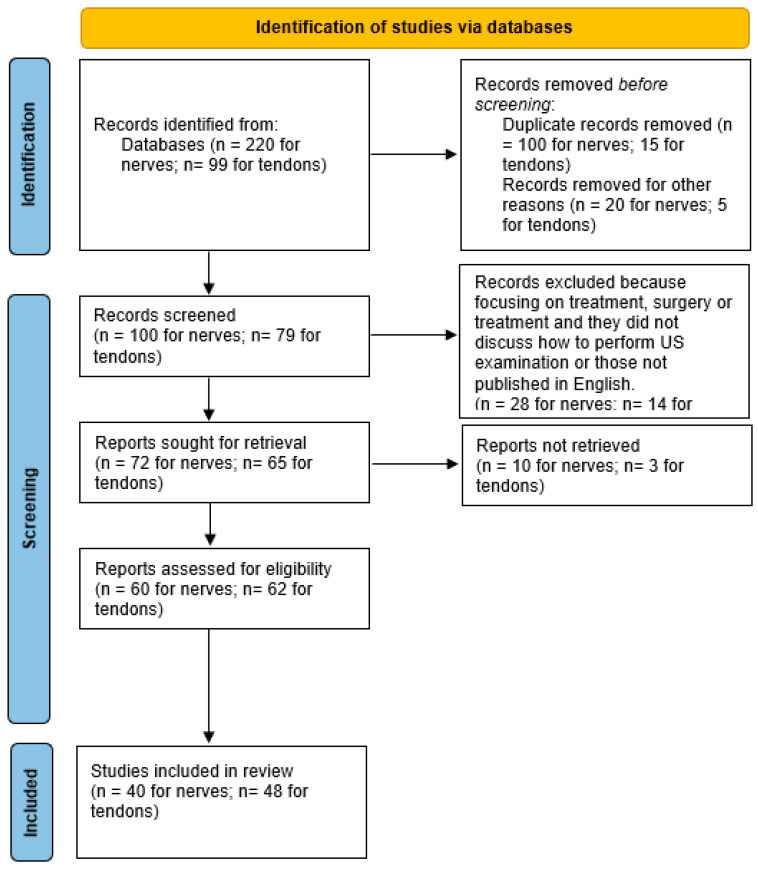
Study selection flow diagram.

The present review was performed according to the Preferred Reporting Items for Systematic reviews and metanalysis (PRISMA). The literature research was carried out using databases like PubMed, Scopus and Web of Science. The following keyword combinations were run: “snapping” OR “popping” OR “dislocation” OR “subluxation” AND “ultrasound imaging” AND/OR “ultrasonography” AND “tendons” OR “nerves”. No publication date or language restrictions were imposed. The initial search yielded 220 papers for nerves and 99 papers for tendons. Thereafter, 120 articles for nerves and 20 for tendons were removed before screening (Figure 1). The retrieved studies (100 papers for nerves and 79 for tendons) were then reviewed. Papers focusing on treatment, surgery or treatment that did not discuss how to perform US examination, or those not published in English, were excluded. A total of 72 papers for nerves and 65 for tendons were further reviewed for their titles and abstracts. Finally, 60 papers for nerves and 62 for tendons were identified for full-text reading, whereby 40 papers for nerves and 48 for tendons were included in this systematic review.

## 3. Results

Papers selected as regards the US imaging of nerve/tendon snapping either in patients or healthy subjects were analyzed. The agreement between the authors for including the articles was perfect (Cohen’s k = 0.87). The main characteristics of the studies (published between 1983 and 2023) are summarized in Table 1 for nerves [6,7,8,9,10,11,12,13,14,15,16,17,18,19,20,21,22,23,24,25,26,27,28,29,30,31,32,33,34,35,36,37,38,39,40,41,42,43,44,45,46] and in Table 2 for tendons [1,47,48,49,50,51,52,53,54,55,56,57,58,59,60,61,62,63,64,65,66,67,68,69,70,71,72,73,74,75,76,77,78,79,80,81,82,83,84,85,86,87,88,89,90,91,92,93,94].

The 40 papers reviewed for nerves comprised 16 original articles, 6 reviews, 14 case reports, 3 retrospective studies and 2 letters to the editor [6,7,8,9,10,11,12,13,14,15,16,17,18,19,20,21,22,23,24,25,26,27,28,29,30,31,32,33,34,35,36,37,38,39,40,41,42,43,44,45,46]. Similarly, 49 papers reviewed for tendons comprised 7 original articles, 17 reviews, 23 case reports/series and 1 retrospective study [48,49,50,51,52,53,54,55,56,57,58,59,60,61,62,63,64,65,66,67,68,69,70,71,72,73,74,75,76,77,78,79,80,81,82,83,84,85,86,87,88,89,90,91,92,93,94]. For nerves, 1156 males (63.8%) and 657 females (36.2%) with an average age of 29.8 ± 15 years had been studied. For tendons, 455 males (29.9%) and 1066 females (70.1%) with an average age of 24.3 ± 14 years had been studied. Usually, the snapping was assessed using B-mode imaging, either with linear or curvilinear probes. The most commonly involved structures were the ulnar nerve (87.5%) and iliopsoas tendon (37.5%).

## 4. Discussion

To the best of our knowledge, this review article is a unique summary of 89 publications on US examination for nerve/tendon snapping. In particular, having also summarized the relevant maneuvers/movements, we have demonstrated the utility of dynamic US imaging as a gold standard diagnostic method for snapping. Of note, this type of assessment not only ascertains the snapping structure, but also the possible abnormalities in relation to the clinical condition [7,8,9,10,11,12,13,14,15,16,17,18,19,20,21,22,23,24,25,26,27,28,29,30,31,32,33,34,35,36,37,38,39,40,41,42,43,44,45,46,47,48,49,50,51,52,53,54,55,56,57,58,59,60,61,62,63,64,65,66,67,68,69,70,71,72,73,74,75,76,77,78,79,80,81,82,83,84,85,86,87,88,89,90,91,92,93,94] (Table 3). 

The etiology of snapping is linked to a wide range of functional factors [1,9,10,34], especially in biomechanical disorders in which the underlying mechanism is complex. Repetitive movements, overuse, muscle and fascial imbalances or structural abnormalities can be reasons for snapping. In some cases, snapping may be painless, while in some others it can be accompanied by significant discomfort/pain. Additionally, patients affected by snapping/popping phenomena are more susceptible to developing chronic pain and limited joint movement [6,7,8,9,10,11,12,13,14,15,16,17,18,19,20,21,22,23,24,25,26,27,28,29,30,31,32,33,34,35,36,37,38,39,40,41,42,43,44,45,46,47,48,49,50,51,52,53,54,55,56,57,58,59,60,61,62,63,64,65,66,67,68,69,70,71,72,73,74,75,76,77,78,79,80,81,82,83,84,85,86,87,88,89,90,91,92,93,94]. The majority of the literature reviewed in this study highlighted the role of US examination to unravel difficulties in diagnosing and unveiling the exact biomechanical alterations associated with snapping/popping due to ambiguous symptoms and signs [1,2,3,4,5]. In this regard, performing a simple US examination following an inconclusive physical examination can undoubtedly be contributory [6,7,8,9,10,11,12,13,14,15,16,17,18,19,20,21,22,23,24,25,26,27,28,29,30,31,32,33,34,35,36,37,38,39,40,41,42,43,44,45,46,47,48,49,50,51,52,53,54,55,56,57,58,59,60,61,62,63,64,65,66,67,68,69,70,71,72,73,74,75,76,77,78,79,80,81,82,83,84,85,86,87,88,89,90,91,92,93,94]. 

Snapping phenomena varied between genders; while the prevalence values were 63.8% (nerves) and 29.9% (tendons) in males, they were, respectively, 36.2% and 70.1% in females. All this could be determined by a different tissue composition in the different sexes. We believe that this ‘almost complete’ opposition needs further investigation.

### 4.1. Nerve Snapping

**Figure 2:**
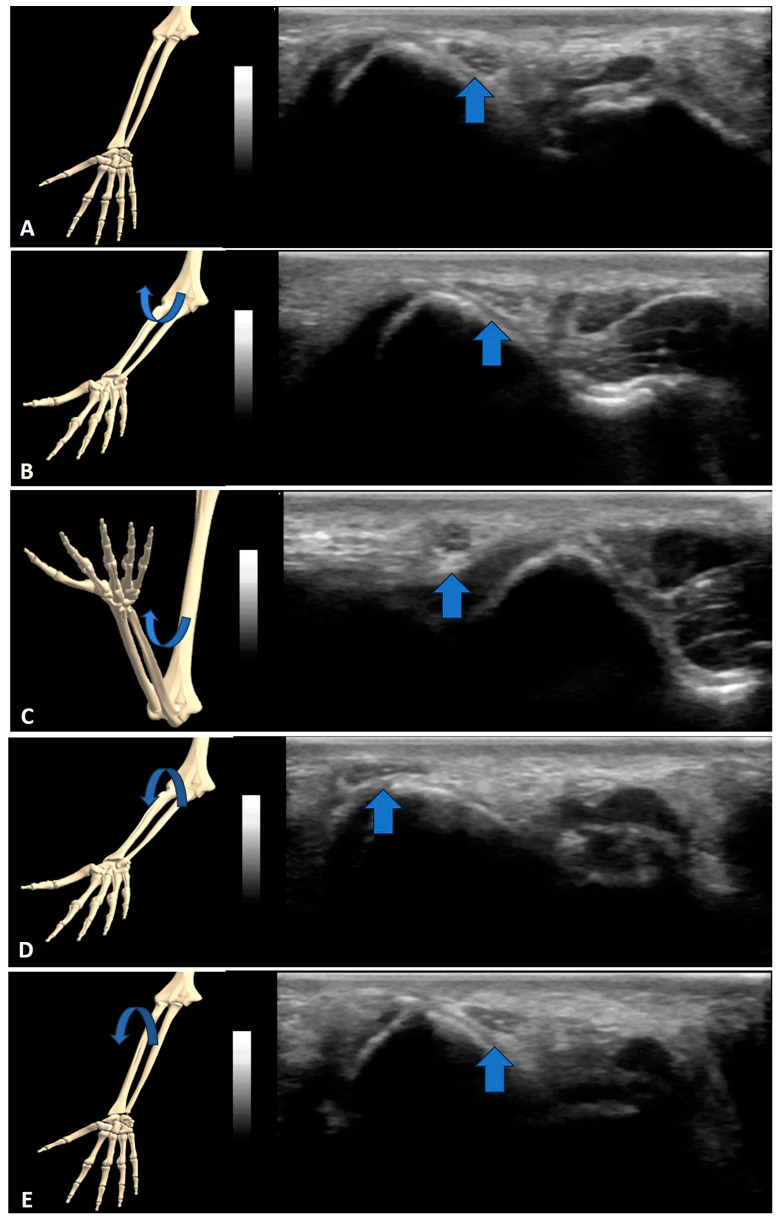
Snapping of the ulnar nerve: (**A**) neutral position, (**B**) 45° elbow flexion, (**C**) 110° elbow flexion, (**D**) 45° elbow flexion during return to neutral position and (**E**) return to neutral position. Arrow: Ulnar nerve.

Concerning nerve snapping (Video S1), the most common regions were reported to be the elbow and the ulnar nerve (87.5%) (Figure 2, Video S2), followed by the medial antebrachial cutaneous nerve (at the elbow), the median nerve (at the wrist) and the sciatic nerve (in the thigh) (Video S3). For the ulnar nerve (snapping over the medial epicondyle), dynamic and short-axis imaging at the cubital tunnel level have been used during various positions of elbow flexion/extension. Additionally, isometric triceps contraction has also been used in certain cases [42].

In this context, considering the benefits of immediate/dynamic visualization of the complete movement, along with the nerve structure along its entire trajectory, dynamic imaging appears to be the most advantageous imaging modality [42,44,47]. Moreover, Shen et al. [44] reported an instability of the ulnar nerve in children, possibly in relation to the flexible retinaculum of the cubital tunnel. Schertz et al. [19] demonstrated that the morphological compression and dislocation of the ulnar nerve correlated with symptomatology. They postulated that patients with anatomic and/or dynamic variation of the ulnar nerve and its surrounding structures were more prone to developing ulnar-nerve-related complaints [19]. Similarly, the snapping of the medial antebrachial cutaneous nerve over the medial epicondyle was assessed during elbow flexion [23]. Median nerve snapping over the palmaris longus tendon [46], the sciatic nerve at the ischiofemoral space (during hip rotations) [28], the proper digital nerve of the fifth toe (during flexion/extension) [29] and the superficial radial nerve during thumb flexion/extension [34] are some other scenarios reported in the literature. Dynamic US examination can be readily performed from a technical standpoint. However, it is imperative for the sonographer to possess detailed knowledge of the local anatomy in order to precisely identify the possible anatomical variations. While such variations can be evaluated (generally statically) by using computed tomography and/or magnetic resonance imaging, US is a far more accessible and affordable diagnostic modality [6,7,8,9,10,11,12,13,14,15,16,17,18,19,20,21,22,23,24,25,26,27,28,29,30,31,32,33,34,35,36,37,38,39,40,41,42,43,44,45,46,47]. It is noteworthy that a visualization of the snapping can also be coupled with the sensation or sound of snapping during real-life instances.

### 4.2. Tendon Snapping

**Figure 3:**
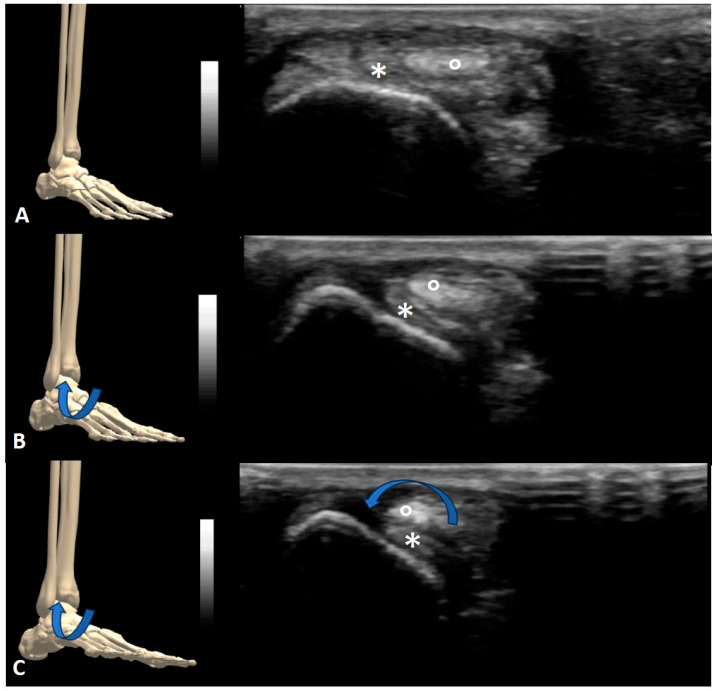
Snapping of peroneal tendons: (**A**) neutral position, (**B**) first degrees of foot eversion and (**C**) complete foot eversion. *: fibularis brevis tendon. °: fibularis longus tendon.

Regarding tendon snapping, several factors such as a conflict with bony structures, other tendons (intersection), retinacula and thickened pulleys, or instability caused by the rupture of retinacula, have been reported [1]. The iliopsoas tendon was the most commonplace (37.5%), in which hip flexion/extension, rotation and abduction were used to induce snapping [50,51,52,64,75,76,79,80,81,93]. Moreover, distal biceps brachii and brachialis tendon snappings [49] were described to ensue (during elbow flexion/extension) over the trochlea. The iliotibial band (during hip flexion/extension) [64], sartorius, gracilis, biceps femoris, popliteus and semitendinosus tendons (during knee flexion/extension), peroneal (Figure 3, Video S4), tibialis posterior and plantaris tendons (during ankle dorsiflexion/inversion), extensor pollicis brevis tendon (during finger flexion/extension), rotator cuff tendons, distal biceps/triceps tendons and wrist flexor/extensor tendons have also been reported to snap in various regions [4,34,68].

Depending on the specific biomechanics of the assessed tendon, combined/detailed positionings of the relevant joints can be easily performed under dynamic US examination [62,78]. For example, biceps femoris snapping usually is shown as a jerky movement of the tendon over the fibular head during knee extension at 90° [1,91]. Magnetic resonance imaging is usually normal in this particular snapping, or may only show a predisposing factor [70,93]. There could possibly be a cord-like anterior arm of the biceps femoris tendon that separates from the direct arm of the tendon 3–4 cm above its insertion [1,91]. Similarly, anatomical variations in other tendons, such as pes anserinus [86], iliopsoas [50,51,52,66,75,76,79,80,81,93], popliteus [73] and peroneals [77,84], can also be predisposed to snapping. In disabling cases, US examination is not only crucial in the diagnosis but also for the eventual pre-operative planning [1].

### 4.3. Future Perspectives Assessing Pros/Cons of Dynamic US Examination in the Evaluation of Nerve/Tendon Snapping 

To date, the concept of dynamic US imaging has been widely accepted [2,3,4]. However, several of the dynamic US assessments obtained can contribute to the exact diagnosis and monitoring of the snapping condition if they are properly interpreted in a clinical/surgical/rehabilitative context. In terms of therapeutic approach, different publications reported that the precise detection of the cause and its severity played an important role. Accordingly, conservative vs. surgical treatment alternatives can be promptly applied, as well as followed, thereafter. Needless to say, the former group includes proper posture maintenance, excessive movement avoidance, regular stretching and strengthening, all of which aim to help muscle/fascial balance and flexibility [95]. 

However, while the pros are that dynamic US imaging enables real-time and multi-directional US observation, providing a more accurate, precise and objective approach to assessing the nerves and tendons movement, the cons would be that in some cases, during dynamic US imaging, it can be difficult to identify which anatomic structure is snapping. Bjerre et al. [39] reported that an accessory snapping triceps tendon can clinically be confused with the snapping of the ulnar nerve [39], as the two structures are closely located at the medial epicondyle. Moreover, a careful evaluation of nearby anatomical structures is mandatory, with particular attention on the various movement directions and degrees during the maneuvers. For example, Asopa et al. [68] demonstrated that the pes anserinus snapping can be secondary to a meniscal cyst, and only by dynamic US imaging was it possible to underline the snapping cause, avoiding incorrect surgery. The MRI revealed a lobulated parameniscal cyst, but it was unable to provide a definitive explanation for the snapping sensation. In contrast, dynamic US imaging permits the successful identification of both meniscal cysts and tears, permitting the observation that the sartorius was anterior to the cyst in the neutral position, while the gracilis tendon was located posteriorly. Inevitably, during knee flexion, the sartorius tendon snapped over the cyst and moved to a posterior position at the front edge of the gracilis tendon. When extending the knee back to a neutral position with active quadriceps muscle contraction, the sartorius tendon swiftly moved forward, traversing over the cyst, resulting in a distressing snapping sensation [68]. 

Due to the superior sensitivity of dynamic US examination in comparison with static US examination and MRI, it has the potential to be an initial modality or to reduce the numbers of imaging examinations. While interest in this US examination is increasing, there are several issues to be considered and solved. First, more methodologically rigorous studies are still needed. The issues in conducting clinical studies include the choice of reference standards for the final diagnosis, the competency of examiners and the standardization of findings. Second, there were few pieces of evidence on the utility of dynamic US examination to differentiate particular nerve/tendon snapping based on the standardization of dynamic US maneuvers. Third, knowledge of anatomical variations is crucial to better highlight the correct anatomical structure snaps and the reasons that determine it. 

The utility of dynamic US examination in nerve/tendon snapping has been shown mainly in the fields of physical and rehabilitative medicine, radiology, orthopedics and neurology. Collaboration between these specialties is indispensable for the further development of this assessment modality.

The limitations of this review would be the small number of patients included in different studies and the heterogeneity of the article types. Also, taking into account the possible variations as regards the expertise of sonographers and the device settings, it was not reasonable or conclusive to carry out further statistical analysis. 

## 5. Conclusions

In closing, this review shows that dynamic US examination can be efficiently incorporated as an extension of physical examination for the evaluation of nerve/tendon snapping in daily clinical practice. It is noteworthy that such an assessment would not only unmask the actual cause/structure responsible for snapping, but would also guide the treatment as well as the close follow up during management. To this end, simultaneously ‘seeing’ and ‘hearing’ the snapping under US examination is invaluable for musculoskeletal physicians.

## Figures and Tables

**Table 1:** Papers on ultrasound and nerve snapping.

Authors and Year	Article Type	Participants	Sex	Age (y)	US Imaging	Maneuver/Movement	Nerve
Cambon-Binder, A. (2021) [7]	Review	-	-	-	B-mode	Elbow flexion/extension	Ulnar
Tsukada, K. et al. (2019) [8]	Retrospective study	246 athletes	M	19.5 ± 1.2 y.	B-mode	Elbow flexion/extension	Ulnar
Pisapia, J.M. et al. (2017) [9]	Case report	1	F	15 y.	B-mode	Elbow flexion/extension	Ulnar
Lee, K.S. et al. (2010) [9]	Review	-	-	-	B-mode	-	Ulnar
Coraci, D. et al. (2017) [10]	Letter to editor	1	F	41 y	B-mode, 18 MHz	45° forearm flexion	Ulnar
Martinoli, C. et al. (1999) [11]	Review	-	-	-	B-mode	Elbow flexion/extension	Ulnar
Kakita, M. et al. (2012) [12]	Clinical trial	38	M	50 ± 15 y.	B-mode	Elbow flexion/extension	Ulnar
Martinoli, C. et al. (2002) [13]	Review	-	-	-	B-mode	Elbow flexion/extension	Ulnar
Omejec, G. et al. (2016) [14]	Original article	226 arms	M	50 ± 14 y.	B-mode	Elbow flexion/extension	Ulnar
Endo, F. et al. (2021) [15]	Original article	153 healthy participants	44 M 112 F	65.4 y.	B-mode	Maximal elbow flexion	Ulnar
Okamoto, M. et al. (2000) [16]	Original article	100 heathy volunteers	50 M 50 F	20–69 y.	B-Mode, 7.5 MHz	Elbow flexion/extension	Ulnar
Cornelson, S.M. et al. (2019) [17]	Case reports	42	25 M 17 F	18–65 y.	B-Mode	Elbow in three different positions: extension, 45° flexion, and full flexion	Ulnar
Kang, S. et al. (2019) [18]	Original article	65	65 M	45 ± 14 y.	B-mode with 5–12 MHz linear array transducer	Elbow full extension to full flexion	Ulnar
Schertz, M. et al. (2017) [19]	Comparative study	117	52 M 65 F	47.3 y.	B-mode with linear probe 5–12 MHz	Starting from 90° flexion to complete flexion of the elbow	Ulnar
Grechenig, W. et al. (2003) [20]	Case reports	2	2 M	38 y. and 12 y.	B-mode	Elbow joint flexion	Ulnar
Kim, B.J. et al. (2008) [21]	Original article	117 healthy volunteers	52 M 65 F	20–50 y.	B-mode with 7.5 to 12 MHz linear transducer.	At any angle during elbow flexion using real-time ultrasonography	Ulnar
Imao, K. et al. (2015) [22]	Case report	1	M	43 y.	B-mode with	During elbow flexion more than 90°	Ulnar
Cesmebasi, A. et al. (2015) [23]	Case report	4	1M 3 F	18.5 y.	B-mode	Snapping over the medial epicondyle	Medial antebrachial cutaneous
Plaikner, M. et al. (2013) [24]	Retrospective study	11	2 M 9 F	28–82 y.	B-mode with linear probe L 17–5 MHz	During maximal extension and flexion of the elbow	Ulnar
Kim, B.J. et al. (2005) [25]	Original article	39	19 M 20 F	20–50 y.	B-Mod with linear probe 7.5 to 12MHz	Elbow extension and flexion	Ulnar
Yoo, M.J. et al. (2007) [26]	Case report	1	F	50 y.	B-Mode with linear probe medium frequency of 10 MHz	At 70 degrees of elbow flexion; at 90 degrees elbow flexion	Ulnar
Shimizu, H. et al. (2011) [27]	Retrospective study	8	4 F 4 M	15–31 y.	B-Mode with linear probe medium frequency of 10 MHz	Elbow flexion/extension	Ulnar
Hatem, M. et al. (2020) [28]	Case report	1	F	64 y.	B-Mode with curvilinear probe	Dislocation from the ischiofemoral space during hip mobilization from internal to external rotation	Sciatic
Reisner, J.H. et al. (2021) [29]	Case series	2	-	-	B-mode	-	Proper Digital of the Fifth Toe
Chuang, H.J. et al. (2016) [30]	Case report	1	M	34 y.	B-mode	During active elbow flexion over 100 degrees	Ulnar
Kang, J.O. et al. (2017) [31]	Original article	26	13 M 13 F	-	B-mode with 13-MHz high-frequency linear array transducer	Elbow in three different positions: extension, 90-degree flexion, and full flexion	Ulnar
Allen, G. et al. (2012) [32]	Review	-	-	-	B-mode	-	Ulnar
Bierre, J.J. et al. (2018) [33]	Case report	2	M	16 y.	B-mode	Elbow flexion/extension	Ulnar
Chang, K.V. et al. (2017) [34]	Case report	1	F	73 y.	B-mode	During extensor, pollicis brevis (EPB) tendon glided over the adjacent abductor pollicis longus (APL) tendon	Superficial Radial
Jacobson, J.A. et al. (2001) [35]	Case report	3	3F	17–52 y.	B-mode with 10-MHz linear transducer	Elbow flexion/extension	Ulnar
Yiannakopoulos, C.K. et al. (2002) [36]	Letter to editors	2	1 F 1 M	28–48 y.	B-mode	-	Ulna
Michael, A.E. et al. (2018) [37]	Cross-sectional study	62	62 M	18–60 y.	B-mode linear array transducer (15–7 MHz)	Cross-section image in elbow extension, 90-degree flexion, maximal flexion, and additionally in maximal flexion with isometric tension of the triceps	Ulnar
Erez, O. et al. (2012) [38]	Prospective study	51	-	6 m.–18 y.	B-mode	Fully extended and flexed past 90 degrees	Ulnar
Granata, G. et al. (2013) [39]	Original article	30	26 F 4 M	15–58 y.	B-mode	Elbow flexion/extension	Ulnar
Tai, T.W. et al. (2014) [40]	Cross-sectional ultrasonographic study	39	M	13 y.	B-mode with 5- to 10-MHz linear-array transducer	Elbow extended and at 45°, 90° and 120° of flexion	Ulnar
Van Den Berg, P.J. et al. (2013) [41]	Prospective study	70	28 M 42 F	19–79 y.	B-mode with a 7–18 MHz linear-array transducer	Patients were positioned supine, keeping the arm beside the head with the elbow flexed to 70 degrees	Ulnar
Kawabata, M. et al. (2022) [42]	Cross-sectional study.	58	56 M 2 F	10–12	B-mode	Elbow flexion/extension	Ulnar
Konin, G.P. et al. (2013) [43]	Review	-	-	-	US B-mode with linear probe of 12–17 MHz	Elbow flexion/extension	Ulnar
Shen, P.C. et al. (2013) [44]	Original article	237	108 F 129 M	6–11 y.	B-mode with a 5 MHz to 10 MHz linear-array transducer	Elbow extended and at 45°, 90° and 120° of flexion	Ulnar
Grechenig, W. et al. (2003) [45]	Case report	2	M	38 y. and 12 y.	B-mode	Elbow extension and flexion	Ulnar
L’Heureux-Lebeau, B. et al. (2012) [46]	Case report	1	M	27 y.	B-mode	Subluxation of the median nerve from one side of the PL tendon during wrist flexion	Median
Filippou, G. et al. (2010) [47]	Original article	91	49 M 42 F	15–81 y.	B-mode	Elbow flexion/extension	Ulnar

y. = years; F = female; M = male; m. = months. - = non specified.

**Table 2:** Papers on ultrasound and tendon snapping.

Authors and Year	Type of Paper	Participants	Sex	Age (y)	US Imaging	Maneuver/Movement	Tendon
Yen, Y.M. et al. (2015) [48]	Review	-	-	-	B-mode	-	Iliopsoas
Ooi, M.W.X. et al. (2022) [49]	Original article	-	-	-	B-mode	Elbow flexion and extension	Distal biceps and brachialis
Lee, K.S. et al. (2013) [50]	Review	-	-	-	B-mode with linear probe 5–12 MHz.	During hip flexion, external rotation, and abduction	Iliopsoas, iliotibial band and gluteus maximus
Janzen, D.L. et al. (1996) [51]	Original article	7	-	17–30 y.	B-mode linear probe 5–12 MHz	During hip flexion, external rotation, and abduction	Iliopsoas
Blankenbaker, D.G. et al. (2008) [52]	Review	-	-	-	B-mode	During hip flexion, external rotation, and abduction	Iliopsoas
Shapiro, S.A. et al. (2017) [53]	Case report	2	1 M 1 F	31 y. and 72 y.	B-mode	Repetitive flexion and extension of knee	Gracilis and semitendinosus
Winston, P. et al. (2007) [54]	Cross-sectional study	87	30 M 57 F	15 to 40 y.	B-mode	The subjects voluntarily reproduced the snap while the hips were scanned	Iliopsoas
Chang, K.V. et al. (2019) [55]	Case report	1	M	42 y.	B-mode	Return from hip flexed and abducted in neutral position; during hip flexion and extension	Iliopsoas
Nolton, E.C. et al. (2018) [56]	Review	-		-	B-mode	During hip flexion and extension	Iliopsoas
Pesquer, L. et al. (2016) [57]	Review	-	-	-	B-mode with high-frequency superficial probes	At different levels of motion in dorsi-flexion, also forced dorsi-flexion	Peroneal
Lungu, E. et al. (2018) [58]	Review	-	-	-	B-mode	During hip flexion and extension	Iliopsoas
Ayhan, E. et al. (2022) [59]	Case report	1	F	18 y.	B-mode with linear probe L14-6 10-MHz	Finger flexion/extension	Extensor pollicis brevis
Draghi, F. et al. (2018) [60]	Review	-	-	-	B-mode with high-frequency	Dorsiflexion	Peroneal
Blankenbaker, D.G. et al. (2006) [61]	Retrospective study	40	15 M 25 F	15–72 y.	B-mode 7–4 MHz; 8–4 MHz, 10 MHz	During hip flexion and extension	Iliopsoas
Allen, G. et al. (2012) [32]	Review	-	-	-	B-mode	-	Rotator cuff, proximal long biceps, distal biceps, rotator cuff, the proximal long head of biceps, the distal biceps, the distal triceps, the flexor and extensor around the elbow and wrist, and the individual within the hand
Erpala, F. et al. (2021) [62]	Prospective randomized study	775	340 M 415 F	18–66 y.	B-mode	Participants were positioned on examination chair with wrist at flexion and forearm at supination (simulating provocation test)	Extensor Carpi ulnaris
Flanum, M.E. et al. (2007) [63]	Case series	6	1 M 5 F	24–48 y.	B-mode	During flexion/extension	Iliopsoas
Chang, K.S. et al. (2015) [64]	Case report	1	M	34 y.	B-mode	Snapping of the ITB over the GT during hip flexion and extension	Iliotibial band
Chang, K.V. et al. (2015) [34]	Case report	1	F	73 y.	B-mode	During extensor, pollicis brevis (EPB) tendon glided over the adjacent abductor pollicis longus (APL) tendon	Extensor pollicis brevis
Piechota, M. et al. (2016) [65]	Review	-	-	-	B-mode	Provocation test	Iliopsoas
Andronic, O. et al. (2019) [66]	Review	-	-	-	B-mode	FABER position, the tendon can be seen snapping over the iliopectineal eminence	Iliopsoas
Blankenbaker, D.G. et al. (2006) [67]	Review	-	-	-	B-mode 5–12 MHz	During flexion	Iliopsoas
Asopa, V. et al. (2013) [68]	Case report	1	M	40 y.	B-mode	Knee flexion/extension	Sartorius
Marchand, A.J. et al. (2012) [1]	Review	-	-	-	B-mode	Knee flexion/extension	Biceps and popliteus
Fantino, O. et al. (2012) [69]	Review	-	-	-	B-mode	Specific tests	Posterior tibialis, peroneal; extensor carpi ulnaris, long head of the biceps muscle
Lohrer, H. et al. (2010) [70]	Review + case report	1	M	58 y.	B-mode	Dislocated posterior tibial tendon over the right malleolus during flexion/extension	Posterior tibialis
Hsieh, T.S. et al. (2019) [71]	Case report	1	F	43 y.	B-mode	During flexion/extension of PIP joint	Extensor digitorum
Greene, B.D. et al. (2021) [72]	Case report	1	F	15 y.	B-mode	Plantar/dorsal flexion	Plantaris
Shukla, D.R. et al. (2014) [73]	Review	-	-	-	B-mode	During flexion/extension	Popliteus
Tanaka, Y. et al. (2015) [74]	Comparative study	24	11 M 13 F	26–74 y.	B-mode	During finger flexion/extension	Flexor digitorum
Anderson, S.A. et al. (2008) [75]	Case series	15	4 M 11 F	15–62 y.	B-mode	During hip flexion/extension	Iliopsoas
Deslandes, M. et al. (2008) [76]	Review and case series	14	5 M 9 F	13–50 y.	B-mode with 5–12 MHz	During hip flexion/extension	Iliopsoas
Raikin, S.M. et al. (2008) [77]	Original article	57	15 M 42 F	-	B-mode	Ankle eversion/inversion	Peroneal
MacLennan, A.J. et al. (2008) [78]	Original article	21	14 M 7 F	14–44 y.	B-mode	Wrist flexion/extension	Extensor carpi ulnaris
Pelsser, V. et al. (2001) [79]	Original article	20	3 M 17 F	12–39 y.	B-mode with curvilinear probe	During hip flexion/extension	Iliopsoas
Cardinal, E. et al. (1996) [80]	Case reports	3	1 M 2 F	24–36 y.	B-mode	During hip flexion/extension	Iliopsoas
de la Hera Cremades, B. et al. (2017) [81]	Case report	1	F	23 y.	B-mode	During hip flexion/extension	Iliopsoas
Han, F. et al. (2014) [82]	Case report	1	M	30 y.	B-mode	During ankle plantar/dorsal flexion	Plantaris
Akagawa, M. et al. (2020) [83]	Case report	1	M	26 y.	B-mode	During knee flexion/extension	Gracilis
Grandberg, C. et al. (2022) [84]	Case report	1	F	25 y.	B-mode	Ankle eversion/inversion	Peroneals
Smith, E. et al. (2022) [85]	Case report	1	F	70 y.	B-mode	Knee flexion/extension	Sartorius
Rainey, C.E. et al. (2015) [86]	Case report	1	M	25 y.	B-mode	Knee flexion/extension	Pes anserinus
Uemura, T. et al. (2021) [87]	Case report	1	M	52 y.	B-mode	Finger flexion/extension	Extensor pollicis brevis
Hung, C.Y. et al. (2018) [88]	Case report	1	M	39 y.	B-mode	Knee flexion/extension	Gracilis
Karataglis, D. et al. (2008) [89]	Case report	1	M	32 y.	B-mode	Knee flexion/extension	Semitendinosus and gracilis
Vidoni, A. et al. (2020) [90]	Case report	1	M	26 y.	B-mode	Finger flexion/extension	Deep flexor digiti
Guillin, R. et al. (2010) [91]	Case report	2	2 M	25–44 y.	B-mode	Knee flexion/extension	Biceps femoris
Martinez-Salazar, E.L.et al. (2018) [92]	Case report	1	F	42 y.	B-mode	Hallux flexion/extension	Flexor hallucis longus
Fazekas, M.L. et al. (2015) [93]	Case report	1	M	14 y.	B-mode	Knee flexion/extension	Semitendinosus and gracilis
Hashimoto, B.E. et al. (1997) [94]	Case report	1	F	14 y.	B-mode	During hip flexion/extension	Iliopsoas

y. = years; M = male; F = female; - = non specified.

**Table 3:** The most common snapping nerves and tendons.

Nerve	Tendon
Ulnar Medial antebrachial cutaneous Sciatic Proper digital (5th toe) Median	Iliopsoas Distal triceps brachii Iliotibial Peroneal Biceps femoris Semitendinosus and gracilis Sartorius Posterior tibialis Extensor pollicis brevis Extensor carpi ulnaris Proximal long biceps brachii Distal long biceps brachii Rotator cuff Deep flexor digiti tendon

## Data Availability

The data presented in this study are available on request from the corresponding author. The data are not publicly available due to privacy.

## Supplementary Materials

The following supporting information can be downloaded at: https://www.mdpi.com/article/10.3390/s23156732/s1, Video S1. Snapping of the radial nerve. Video S2. Snapping of the ulnar nerve. Video S3. Snapping of the sciatic nerve. Video S4. Snapping of the peroneal tendons.
